# Metabolomics in severe traumatic brain injury: a scoping review

**DOI:** 10.1186/s12868-023-00824-1

**Published:** 2023-10-16

**Authors:** Riley Page Fedoruk, Chel Hee Lee, Mohammad Mehdi Banoei, Brent W. Winston

**Affiliations:** 1 Department of Critical Care, Cumming School of Medicine, Alberta Health Services and University of Calgary, Calgary, Canada; 2 Department of Mathematics and Statistics, Faculty of Science, University of Calgary, Calgary, Canada; 3 Department of Biological Sciences, University of Calgary, Calgary, Canada; 4 Departments of Medicine and Biochemistry and Molecular Biology, Cumming School of Medicine, University of Calgary, Calgary, Canada

**Keywords:** Traumatic brain injury (TBI), Severe traumatic brain injury (sTBI), Metabolomics, Scoping review

## Abstract

**Background:**

Diagnosis and prognostication of severe traumatic brain injury (sTBI) continue to be problematic despite years of research efforts. There are currently no clinically reliable biomarkers, though advances in protein biomarkers are being made. Utilizing Omics technology, particularly metabolomics, may provide new diagnostic biomarkers for sTBI. Several published studies have attempted to determine the specific metabolites and metabolic pathways involved; these studies will be reviewed.

**Aims:**

This scoping review aims to summarize the current literature concerning metabolomics in sTBI, review the comprehensive data, and identify commonalities, if any, to define metabolites with potential clinical use. In addition, we will examine related metabolic pathways through pathway analysis.

**Methods:**

Scoping review methodology was used to examine the current literature published in Embase, Scopus, PubMed, and Medline. An initial 1090 publications were identified and vetted with specific inclusion criteria. Of these, 20 publications were selected for further examination and summary. Metabolic data was classified using the Human Metabolome Database (HMDB) and arranged to determine the ‘recurrent’ metabolites and classes found in sTBI. To help understand potential mechanisms of injury, pathway analysis was performed using these metabolites and the Kyoto Encyclopedia of Genes and Genomes (KEGG) Pathway Database.

**Results:**

Several metabolites related to sTBI and their effects on biological pathways were identified in this review. Across the literature, proline, citrulline, lactate, alanine, valine, leucine, and serine all decreased in adults post sTBI, whereas both octanoic and decanoic acid increased. Hydroxy acids and organooxygen compounds generally increased following sTBI, while most carboxylic acids decreased. Pathway analysis showed significantly affected glycine and serine metabolism, glycolysis, branched-chain amino acid (BCAA) metabolism, and other amino acid metabolisms. Interestingly, no tricarboxylic acid cycle metabolites were affected.

**Conclusion:**

Aside from a select few metabolites, classification of a metabolic profile proved difficult due to significant ambiguity between study design, sample size, type of sample, metabolomic detection techniques, and other confounding variables found in sTBI literature. Given the trends found in some studies, further metabolomics investigation of sTBI may be useful to identify clinically relevant metabolites.

**Supplementary Information:**

The online version contains supplementary material available at 10.1186/s12868-023-00824-1.

## Introduction

### Traumatic brain injury

Traumatic Brain Injury (TBI) is slowly becoming one of the leading causes of death and disability worldwide [1–3]. Currently, about half the world’s population is expected to experience a TBI within their lifetime [4], and by the year 2031, TBI is anticipated to be one of the most common neurological conditions affecting the globe [5, 6]. TBI is defined as a sudden external trauma to the head causing both immediate and delayed alterations to brain function [7]. Following primary injury, secondary injury mechanisms such as cerebral edema, hypoxia, and subarachnoid hemorrhage continue to disrupt the brain’s cells and tissues, causing further damage [8–11]. There are three clinically defined severity levels of TBI based on the Glasgow Coma Scale (GCS): mild traumatic brain injury (mTBI, GCS 13-15), moderate traumatic brain injury (moTBI, GCS 9-12), and severe traumatic brain injury (sTBI, GCS 3-8). sTBI is defined as having a Glasgow Coma Scale (GCS) of 3-8, which is assessed by examining verbal, eye, and motor responses in an individual suspected of having a brain injury [12]. Understandably, sTBI causes the largest economic and societal strains on health care systems as individuals are often unresponsive, or even comatose, require life support, and can have significant residual effects if they survive [13, 14]. A multicenter study by Dawes and colleagues in 2015 found that unadjusted mortality rates varied from 20 to 50% in adults with sTBI (GCS < 9) [15]. Furthermore, the dynamic nature of sTBI pathology can lead to unfortunate misdiagnoses and misinterpretations of severity [16], especially in polytrauma patients. sTBI is a chronic disease process and should be treated as such; the life-long repercussions of these injuries can severely impact an individual's life expectancy and quality of life [17].

Current clinical assessment techniques for sTBI include Computed Tomography (CT) Scans and Magnetic Resonance Imaging (MRI). While these techniques excel in diagnosing the injury, they lack the required specificity to recognize the severity and make accurate outcome predictions for the entire range of sTBI injuries. Additionally, sedation and analgesia can interfere with diagnosis and outcome assessments, especially in patients suffering polytrauma associated with TBI. Therefore, new reliable diagnostic and prognostic methods are needed for more accurate sTBI diagnosis and prognosis.

### Metabolomics as a diagnostic and prognostic tool

Metabolomics is a diagnostic tool used to identify metabolites within cells, tissues, and fluids of biological organisms. It is categorized under the ‘Omics’ line of health research technologies along with genomics and proteomics which all focus on identifying, characterizing, and quantifying the biological molecules involved in the structural and functional organization of organisms [18, 19]. Metabolomics focuses on the classification of metabolites within the human metabolome to help gain insight into the pathophysiological processes of various illnesses and diseases. ‘Biomarker’ is the term given to metabolites or characteristics that are recognized indicators of change in biological processes, such as those defining the pathological mechanisms of sTBI [20]. Currently, there are no widely accepted metabolite biomarkers for sTBI. However, the identification of clinically relevant biomarkers could potentially lead to the creation of novel therapeutics, more accurate diagnoses of disease severity, and more reliable prognostication. This could have an immense impact on the treatment protocols for sTBI and support the ongoing research to identify specific metabolites (biomarkers) involved in sTBI pathogenesis.

Metabolic analysis uses a wide array of different sampling methods. Serum and plasma are among the most frequently used sample types, as they are typically the easiest to retrieve from injured individuals. However, collection sites for serum and plasma are generally at least one compartment away from the injured brain, allowing for the interference of confounders, such as compensatory mechanisms and polytrauma injuries, specifically in sTBI patients. Cerebrospinal Fluid (CSF) as well as brain microdialysate can also be used in metabolomics analysis and appears to provide the most precise measurements due to proximity to the injured area. However, CSF retrieval requires invasive methods such as lumbar punctures, and for this reason, they are obtained much less often in TBI. Additionally, sources of metabolomics analysis such as urine, feces, and magnetic resonance imaging spectroscopy (MRIS) have been explored in metabolite determination for TBI in humans [21].

Once samples have been retrieved, several different analytical platforms can be utilized for the identification and quantification of metabolites. Metabolite measurement is divided into two different approaches, targeted and non-targeted. Targeted metabolomics is the identification and quantification of previously defined and chemically characterized metabolites, while untargeted metabolomics is the overall general identification and relative quantification of all measurable analytes and metabolites in a sample, including those that are unknown [22]. The most frequently used analytical approaches for measuring metabolites include gas chromatography-mass spectrometry (GC–MS), liquid chromatography-mass spectrometry (LC–MS), and proton nuclear magnetic resonance (^1^H-NMR) spectroscopy. MRIS is being used more frequently on the brain as a non-invasive method for identifying metabolites in tissues using sophisticated MRI techniques; however, using current technology, resolution, and metabolite identification are limited [23].

### Metabolomics in severe traumatic brain injury

The purpose of this scoping review is to summarize the current literature on metabolomics investigations in sTBI and their metabolic data to determine if any common metabolite patterns exist. Aside from a study published in 2017 by Posti and colleagues [16], a scoping review of this kind, comparing results across a large selection of adult human metabolomic studies with the addition of affected pathways, has yet to be completed, to our knowledge. An updated and comprehensive summary of the current literature on metabolomics in sTBI is needed to determine clinically reliable biomarkers.

This scoping review encompasses most of the recent metabolite literature published for sTBI in adult cohorts. However, there is unfortunately a general lack of clarity between metabolic studies due to differing sampling methods, sample types, analytical techniques, and study designs. This study aims to dissect the differences between the studies and determine if there are any commonalities or overall patterns apparent in metabolites following sTBI. It is hoped that the identification of reliable metabolites as ‘biomarkers’ may be the answer to providing a more precise diagnosis and prognosis for all severities of TBI, especially sTBI. The accumulation of data in this review could aid in the creation of a generalized metabolic profile or provide important clues about potential biomarkers for sTBI in adult cohorts.

## Methods

This Scoping review was conducted following a modified version of the *Preferred Reporting Items for Systematic Reviews and Meta-Analyses Extension for Scoping Reviews* (PRISMA-ScR) [24]. Four bibliographic databases were explored in this scoping review: CINAHL, Ovid Medline, PubMed, and Scopus. Hand searched articles from select reference lists and the University of Calgary library were also included.

### Search strategy

The research goal was to comprehensively gather published literature since the year 2000 on metabolomics in severe traumatic brain injury among adults and create a profile of recurrent metabolites and related pathways that could support clinical applications. A key concept chart was created including sets of search terms to be explored, such as “metabolomics in sTBI”, “data analysis” and, “biomarkers in sTBI”, as well as potential free text terms like “metabolites”, “severe traumatic brain injury” and “adult cohort”. Search phrases were then created using free text terms and Boolean operators such as OR/AND where appropriate. The main phrase chosen to search the databases was “metabolomics” AND “severe traumatic brain injury”. Additional phrases, including “biomarkers” AND "severe traumatic brain injury,” were also applied in the primary search. Further hand searching revealed several more articles which were also included in the study.

**Fig. 1:**
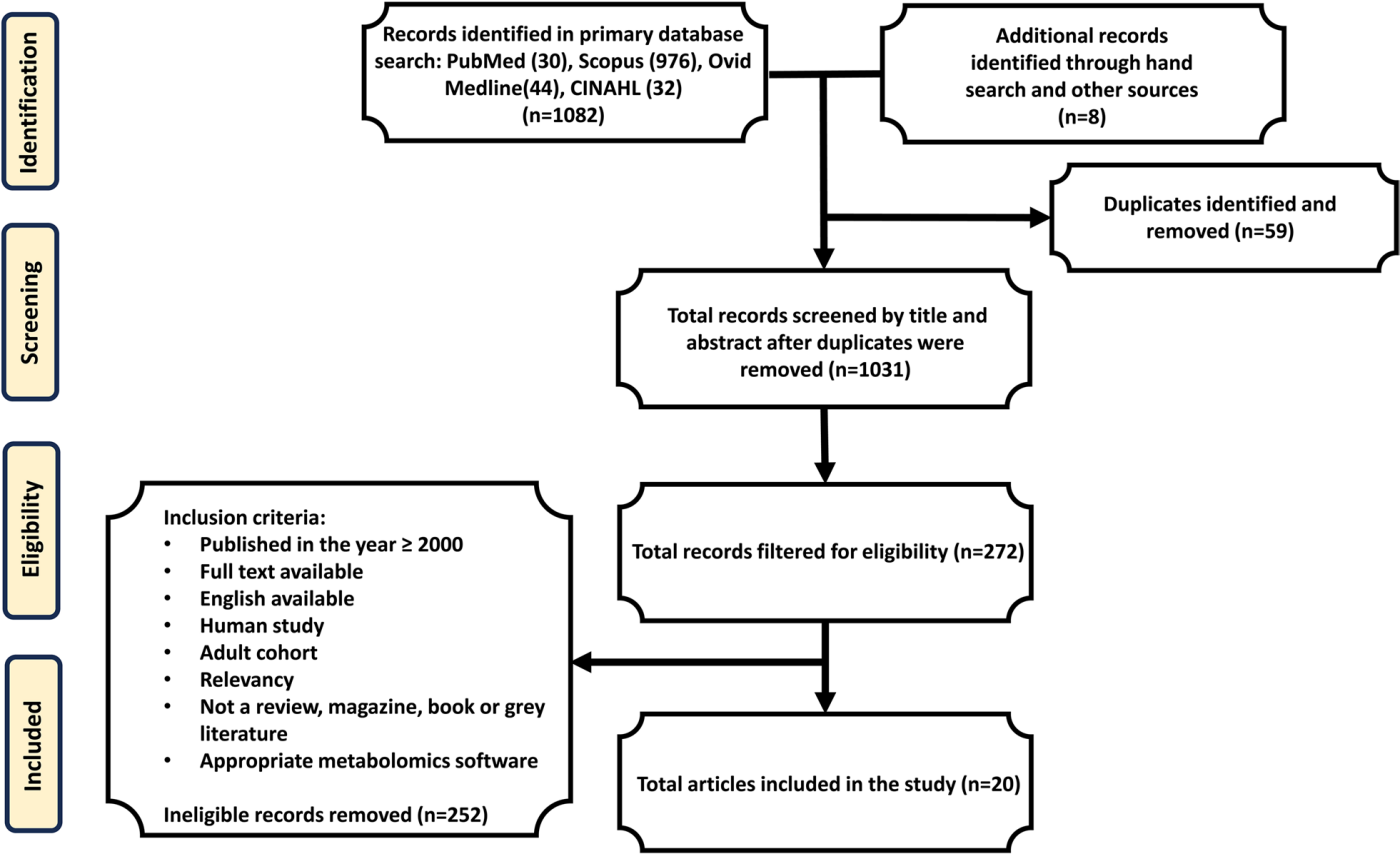
Consort Diagram for the publication selection process

The database search retrieved 1082 publications in total; PubMed (n = 30), Scopus (n = 976), Medline Ovid (n = 44), and CINAHL (n=32), and 8 additional hand searched references (see Fig. 1, consort diagram). The publications were combined in *Clarivate Analytics: EndNote*, a referencing software, and 59 duplicates were removed, resulting in 1031 publications qualifying for the screening process. Based on the primary search, abstracts, and titles were screened and eliminated if deemed irrelevant. Screening greatly reduced the number of publications to 272. Eligibility was then decided using previously established inclusion criteria for the remaining publications. The included articles were published in or after the year 2000—with an emphasis on publications within the last decade, full text, and availability in English. Results were then filtered for human studies with adult cohorts and appropriate metabolomics software analysis. Literature was not included if classified as a review, grey literature, magazine, or book. Additional studies were later removed for irrelevancy, other types of TBI not including sTBI, and lack of clarity in results. The eligibility process yielded 20 suitable publications for metabolomics investigations in sTBI to be summarized in this scoping review.

### Data collection and classification

The included publications were reviewed in full and comprehensive summary tables were produced using Microsoft Excel. Publications were summarized using headings such as year, author, sample type and size, detection technique, major metabolite findings, and other varying statistics. A subsequent table was generated using the major metabolite findings and incorporated a more thorough breakdown of the exact metabolites found in each study and whether they increased, decreased, or were unchanged. In this analysis, ‘recurrent’ metabolites were defined as any metabolite that was found either increasing or decreasing in two or more of the included studies and was therefore determined to have a stronger connection to sTBI in adults. All metabolites were then further analyzed through organization by class using the Human Metabolome Database (HMDB). The Class, Subclass, Super Class, and HMDB code of each metabolite were included in the initial summary table. The format of these tables allowed for the direct comparison and visualization of specific metabolites and patterns between studies.

### Pathway analysis

A pathway analysis diagram was then manually generated using the major metabolite findings and the Kyoto Encyclopedia of Genes and Genomes (KEGG) Pathway Database. The diagram was produced to demonstrate the major pathways affected by sTBI pathogenesis. Once specific pathways were identified, further literature analysis was conducted to expand on the results. Emphasis was placed on those showing the same metabolites affected in more than one study.

## Results

The present scoping review analyzed 20 publications with established investigations of metabolomics in severe traumatic brain injury among adults. A summary (Additional file 1: Table S1) is provided in the Additional file for the context and setting of the examined publications.

Many of the explored studies undertook broad metabolic analysis in adult cohorts searching for overall metabolite alterations post TBI, while a select few specified on individual metabolites. For example, a study by Marino and colleagues used proton nuclear magnetic resonance imaging (^1^H-MRIS) to examine metabolic levels of N-acetylaspartate (NAA), choline, creatine, and lactate following brain injury [25], whereas Jeter and colleagues utilized LC–MS and GC–MS to measure L-arginine levels and branched chain amino acids (BCAA) in patients post TBI [26, 27]. Another study examined extracellular NAA in microdialysate using high-performance liquid chromatography (HPLC) [28]. Methionine alteration post TBI was investigated by Dash and colleagues in 2016 using LC–MS and GS-MS [29]. More recent literature includes work by Bykowski and coworkers who utilized ^1^H-NMR technology in urine to determine changes in metabolites during TBI recovery and the correlation to injury severity [30]. Earlier this year, Mondello and his team published a study that used LC–MS to investigate serum glycome patterns following TBI [31] and another study used Center-TBI data to describe the human metabolome associated with TBI [32]. A more detailed breakdown of the reviewed studies can be found in Table A1.

**Table 1:** 15 recurrent metabolites (bolded) found indicative of stronger connection to severe traumatic brain injury pathology

Metabolite	Sample type	Change identified	Supplemental	Refs.
Octanoic acid				
	Serum/Plasma	Increase	Increased as TBI severity increased	Thomas et al. [32]
	Serum/Microdialysate	Increase	Found upregulated in sTBI patients and remained high in most patients	Oresic et al. [33]
Decanoic acid				
	Serum/Plasma	Increase	Increased as TBI severity increased	Thomas et al. [32]
	Serum/Microdialysate	Increase	Found upregulated in sTBI patients and remained high in most patients	Oresic et al. [33]
Serine				
	Serum/Microdialysate	Decrease	Found downregulated in all TBIs and more strongly in sTBI	Oresic et al. [33]
	Serum/Plasma	Decrease	Found decreased in all TBI patients overall	Thomas et al. [32]
	Serum/Plasma	Decrease	≤ 60% the concentration in jugular blood compared to HC	Wolahan et al. [34]
Inositol/Myo-inositol				
Inositol	Serum/Microdialysate	Increase	Found to increase in patients with detectable pathology on a CT scan or the presence of a mass lesion following TBI	Dickens et al. [36]
Myo-inositol	Serum/Plasma	Increase	Found elevated in TBI patients and proportional to differing severity	Thomas et al. [32]
Alanine				
	Serum/Microdialysate	Decrease	Found downregulated in all TBIs and more strongly in sTBI	Oresic et al. [33]
	Serum/Plasma	Decrease	Found decreased in TBI patients overall	Thomas et al. [32]
Choline				
	Serum/Plasma	Decrease	Significantly reduced in sTBI compared to mTBI and healthy volunteers	Dash et al. [29]
	Serum/Plasma	Increase	Net cerebral release or increase in jugular venous blood	Wolahan et al. [34]
	MR imaging	Increase	Decrease in patients with differing degrees of TBI, including sTBI	Marino et al. [25]
Lactate				
	CSF	Increase	A slight statistically increasing trend in sTBI patients compared to non-injured controls	Glenn et al. [37]
	MR Imaging	Increase	Diffusely high signal of lactate resonance intensity in patients with acute TBI including sTBI	Marino et al. [25]
	CSF	Increase	Significantly increased compared to control and survival groups	Stefani et al. [38]
Citrulline				
	Serum/Plasma	Decrease	≤ 60% the concentration in jugular blood compared to HC	Wolahan et al. [34]
	Serum/Plasma	Decrease	Significantly reduced in the plasma of sTBI patients	Jeter et al. [26]
Proline and derivatives				
Proline	Serum/Plasma	Decrease	≤ 60% the concentration in arterial plasma compared to HC	Wolahan et al. [34]
Proline	Serum/Plasma	Decrease	Significantly reduced in the plasma of sTBI patients compared to healthy volunteers, orthopedic controls, and mTBI patients	Jeter et al. [26]
5-oxoproline	Serum/Plasma	Decrease	Significantly reduced in sTBI patients compared to mTBI and healthy volunteers	Dash et al. [29]
Hydroxyproline	Serum/Plasma	Decrease	Significantly reduced in the plasma of sTBI patients compared to healthy volunteers, orthopedic controls, and mTBI patients	Jeter et al. [26]
Hydroxyproline	Serum/Plasma	Decrease	≤ 60% the concentration in jugular blood compared to HC	Wolahan et al. [34]
Methionine				
	Urine	Increase	Significant negative correlation (increase) to patients decreasing GCS scores	Bykowski et al. [30]
	Serum/Plasma	Decrease	Significant reduction in plasma relative to healthy volunteers	Dash et al. [29]
Xanthine				
	Serum/Plasma	Increase	Significant net cerebral release/increase in jugular venous blood	Wolahan et al. [34]
	Urine	Increase	Increased levels following recovery	Bykowski et al. [30]
N-acetylaspartate				
	Microdialysate	Decrease	Steep decline of extracellular NAA seen in 8 patients early on	Shannon et al. [28]
	MR Imaging	Decrease	Decrease in patients with differing degrees of TBI, including sTBI	Marino et al. [25]
2/3-hydroxybutryic acid				
2-hydroxybutyrate	Serum/Plasma	Increase	Found significantly increased in sTBI patients	Dash et al. [29]
2-hydroxybutyric acid	Serum/Microdialysate	Increase	Found upregulated in sTBI patients	Oresic et al. [33]
3-hydroxybutyric acid	Serum/Microdialysate	Increase	Found upregulated in sTBI patients	Oresic et al. [33]
Valine				
	Serum/Plasma	Decrease	Reduction in levels detected in the plasma of sTBI patients compared to all other groups	Jeter et al. [27]
	Serum/Plasma	Decrease	Significantly decreased during the first week post Stbi compared to controls	Vuille-Dit-Bille [35]
Leucine				
	Serum/Plasma	Decrease	Decrease in patients with sTBI compared to healthy volunteers and mTBI patients	Jeter et al. [27]
	Serum/Plasma	Decrease	Significantly decreased during the first week post sTBI compared to controls	Vuille-Dit-Bille [35]

*CT* computed tomography; *GCS* Glasgow Coma Scale; *HC* healthy controls; *mTBI* minor traumatic brain injury; *NAA* N-acetylaspartate; *sTBI* severe traumatic brain injury; *TBI* traumatic brain injury

### Fifteen ‘recurrent’ metabolites were identified across publications

In this review, a metabolite was defined as ‘recurrent’ if it was found either increasing or decreasing in two or more of the included studies and was therefore determined to have a stronger connection to sTBI. Fifteen ‘recurrent’ metabolites were identified in this analysis and are displayed below [Table 1], along with their respective references, methods for sample collection, and supplemental information. The collected data shows that two medium-chain fatty acids, octanoic and decanoic acid, both increase in adults after sTBI [32, 33]. The data also displays an increase in 2- and 3-hydroxybutyric acids following sTBI [29, 33].

Furthermore, this review found that after sTBI, serine [32–34], alanine [32, 33], proline [26, 29, 34], valine [27, 35], and leucine [27, 35] all decreased, while conflicting results were found for methionine [29, 30]. Choline presented conflicting results as well, but more studies declared an increase in choline following sTBI [25, 29, 34]. Inositol [36] and myo-inositol [32] were also found to increase after sTBI, while n-acetylaspartate [25, 28] and citrulline [26, 34] were found to decrease. Finally, an increase in both xanthine [30, 34] and lactate [25, 37, 38] was found following sTBI in adults. These metabolites represent findings supported by more than one study and thus may serve as a preliminary metabolic profile of sTBI in adults. However, it is important to consider the limitations of combining primary data from several studies to reach conclusions, which will be addressed in the discussion below.

### Classification of all metabolites by human metabolome database identified clear alterations in metabolite groups

To further analyze and specify metabolic alterations following sTBI in adults, the collected metabolite data was categorized using the Human Metabolome Database (HMDB). For this analysis, all metabolite data was included, together with the ‘recurrent’ metabolites. Thirteen different metabolite classes were identified from the collection of data. Table 2 presents the metabolites arranged by class along with their respective references, sample collection method, and supplemental information. Summarization of the literature displayed that after sTBI, carboxylic acids and derivatives primarily decreased. However, several exceptions existed, including creatine, glutamine, phenylalanine, methionine, glutamate, and tyrosine, which all increased. Following sTBI, a consistent increase was seen in hydroxy acids and derivatives. Aside from an elevation in octanoic and decanoic acids, fatty acyls were found to primarily decrease. Organooxygen compounds mostly increased following sTBI, aside from a select few glucose derivatives which decreased. Both purine nucleosides and imidazopyrimidines exhibited increasing trends. Adversely, keto acids and derivatives and organonitrogens both presented inconsistent results between classified metabolites, rendering conclusions difficult. Other classes with trivial amounts of metabolites were also identified but were not deemed significant enough to warrant any major conclusions.

**Table 2:** Human metabolome database (HMDB) classification of collected metabolites by class (bolded)

HMDB class	Metabolite	Sample type	Change identified	Supplemental	Refs.
Fatty acyls					
	Isobutyrylcarnitine	Serum/Plasma	Decrease	Significant decrease in sTBI patients compared to healthy volunteers, orthopedic patients and mTBI	Jeter et al. [27]
	Isoleucine	Serum/Plasma	Decrease	Significant decrease in sTBI patients compared to healthy volunteers, orthopedic patients and mTBI	Jeter et al. [27]
	Propionylcarnitine	Serum/Plasma	Decrease	Decent reduction in sTBI patients compared to healthy volunteers	Jeter et al. [27]
	Succinylcarnitine	Serum/Plasma	Decrease	Only differed by a decrease in sTBI patients compared to orthopedic injury patients	Jeter et al. [27]
	Hydroxyisovaleryl carnitine	Serum/Plasma	Decrease	Found decreased exclusively in sTBI patients compared to healthy volunteers	Jeter et al. [27]
	Octanoic acid	Serum/Microdialysate	Increase	Found upregulated in sTBI patients and remained high in most patients	Oresic et al. [33]
	Decanoic acid	Serum/Microdialysate	Increase	Found upregulated in sTBI patients and remained high in most patients	Oresic et al. [33]
	Octanoic acid	Serum/Plasma	Increase	As TBI severity increased, octanoic acid increased	Thomas et al. [32]
	Decanoic acid	Serum/Plasma	Increase	As TBI severity increased, decanoic acid increased	Thomas et al. [32]
	2-methylbutyrylcarnitine	Serum/Plasma	Decrease	Significant decrease in sTBI patients compared to healthy volunteers and mTBI	Jeter et al. [27]
	Isovalerylcarnitine	Serum/Plasma	Decrease	Significant decrease in sTBI patients compared to healthy volunteers, orthopedic controls, and mTBI	Jeter et al. [27]
Keto acids and derivatives					
	α-ketobutyrate	Serum/Plasma	Increase	Found significantly increased in the plasma of sTBI patients	Dash et al. [29]
	Methylglutarylcarnitine	Serum/Plasma	Increase	Plasma levels were found significantly increased in sTBI patients compared to healthy volunteers, orthopedic controls, and mTBI	Jeter et al. [27]
	4-methyl-2-oxopentanoate	Serum/Plasma	Decrease	Decreased in sTBI patients compared to healthy volunteers	Jeter et al. [27]
	3-methyl-2-oxovalerate	Serum/Plasma	Decrease	Significant decrease in sTBI patients compared to healthy volunteers, orthopedic patients, and mTBI	Jeter et al. [27]
Carboxylic acids and derivatives					
	Methionine	Serum/Plasma	Decrease	Significant reduction in plasma relative to healthy volunteers	Dash et al. [29]
	Betaine	Serum/Plasma	Decrease	Significantly reduced compared to mTBI group and healthy volunteers	Dash et al. [29]
	Dimethylglycine	Serum/Plasma	Decrease	Significant decrease in sTBI patients relative to healthy volunteers	Dash et al. [29]
	Cysteine	Serum/Plasma	Decrease	Showed a significant reduction in sTBI patients	Dash et al. [29]
	Glycine	Serum/Plasma	Decrease	Significantly reduced in sTBI and mTBI	Dash et al. [29]
	Gamma-glutamylvaline	Serum/Plasma	Decrease	Relative levels were found significantly decreased in sTBI patients	Dash et al. [29]
	Gamma-glutamylleucine	Serum/Plasma	Decrease	Relative levels were found significantly decreased in sTBI patients	Dash et al. [29]
	Gamma-glutamylisoleucine	Serum/Plasma	Decrease	Relative levels were found significantly decreased in sTBI patients	Dash et al. [29]
	Gamma-glutamyltyrosine	Serum/Plasma	Decrease	Relative levels were found significantly decreased in sTBI patients	Dash et al. [29]
	Gamma-glutamylphenylalanine	Serum/Plasma	Decrease	Relative levels were found significantly decreased in sTBI patients	Dash et al. [29]
	5-oxoproline	Serum/Plasma	Decrease	Significantly reduced in sTBI patients compared to mTBI and healthy volunteers	Dash et al. [29]
	2-Aminobutyric acid	Serum/Plasma/Microdialysate	Decrease	Found lower concentrations in patients with detectable CT features following TBI	Dickens et al. [36]
	Citrulline	Serum/Plasma	Decrease	Significantly reduced in the plasma of sTBI patients	Jeter et al. [26]
	Ornithine	Serum/Plasma	Decrease	Significant decrease in the plasma of sTBI patients compared to healthy volunteers, orthopedic controls, and mTBI patients	Jeter et al. [26]
	Proline	Serum/Plasma	Decrease	Significantly reduced in the plasma of sTBI patients compared to healthy volunteers, orthopedic controls, and mTBI patients	Jeter et al. [26]
	4-Hydroxyproline	Serum/Plasma	Decrease	Significantly reduced in the plasma of sTBI patients compared to healthy volunteers, orthopedic controls, and mTBI patients	Jeter et al. [26]
	Creatine	Serum/Plasma	Increase	Significantly increased in sTBI patients compared to HC and orthopedic controls	Jeter et al. [26]
	Valine	Serum/Plasma	Decrease	Significantly decreased during the first week post sTBI compared to controls	Vuille-Dit-Bille [35]
	Valine	Serum/Plasma	Decrease	Reduction in valine levels detected in the plasma of sTBI patients compared to all other groups	Jeter et al. [27]
	Serine	Serum/Microdialysate	Decrease	Found downregulated in all TBIs, more strongly in sTBI	Oresic et al. [33]
	Leucine	Serum/Plasma	Decrease	Significantly decreased during the first week post sTBI compared to controls	Vuille-Dit-Bille [35]
	Isoleucine	Serum/Plasma	Decrease	Significantly decreased during the first week post sTBI compared to controls	Vuille-Dit-Bille [35]
	Leucine	Serum/Plasma	Decrease	Decrease in patients with sTBI compared to healthy volunteers and mTBI patients	Jeter et al. [27]
	Alanine	Serum/Microdialysate	Decrease	Found downregulated in all TBIs, more strongly in sTBI	Oresic et al. [33]
	Glutamine	CSF	Increase	Statistically increasing trend in moTBI and sTBI patients compared to non-injured controls	Glenn et al. [37]
	Creatinine	CSF	Decrease	Significantly decreased concentrations of total creatinine in moTBI and sTBI patients	Glenn et al. [37]
	Proline	Serum/Plasma	Decrease	≤ 60% the conc. in arterial plasma and jugular blood compared to HC	Wolahan et al. [34]
	Hydroxyproline	Serum/Plasma	Decrease	≤ 60% the conc. in jugular blood compared to HC	Wolahan et al. [34]
	Serine	Serum/Plasma	Decrease	≤ 60% the conc. in jugular blood compared to HC	Wolahan et al. [34]
	Citrulline	Serum/Plasma	Decrease	≤ 60% the conc. in jugular blood compared to HC	Wolahan et al. [34]
	eNAA	Microdialysate	Decrease	Steep decline of extracellular NAA seen in 8 patients early on	Shannon et al. [28]
	Phenylalanine	Serum/Plasma	Increase	Significantly increased during the first posttraumatic week following TBI	Vuille-Dit-Bille [35]
	Methionine	Urine	Increase	Significant negative correlation (increase) to patients decreasing GCS scores	Bykowski et al. [30]
	NAA	MR Imaging	Decrease	Decrease in patients with differing degrees of TBI, including sTBI	Marino et al. [25]
	L-arginine	Serum/Plasma	Decrease	Significant reduction compared to healthy volunteers, orthopedic injury patients, and mTBI	Jeter et al. [26]
	Serine	Serum/Plasma	Decrease	Found decreased in TBI patients overall	Thomas et al. [32]
	Alanine	Serum/Plasma	Decrease	Found decreased in TBI patients overall	Thomas et al. [32]
	Glutamate	CSF	Increase	Significantly increased compared to control and survival groups	Stefani et al. [38]
	Tyrosine	Serum/Plasma	Increase	Significantly increased for both posttraumatic weeks following TBI	Vuille-Dit-Bille [35]
	Asparagine	Serum/Plasma	Decrease	TBI blood had less than or equal to 60% the concentration of HC in jugular blood	Wolahan et al. [34]
	Threonine	Serum/Plasma	Decrease	Found decreased in TBI patients overall	Thomas et al. [32]
Hydroxy acids and derivatives					
	2-hydroxybutyrate	Serum/Plasma	Increase	Found significantly increased in sTBI	Dash et al. [29]
	2-hydroxybutyric acid	Serum/Microdialysate	Increase	Found upregulated in sTBI patients	Oresic et al. [33]
	3-hydroxybutyric acid	Serum/Microdialysate	Increase	Found upregulated in sTBI patients	Oresic et al. [33]
	Lactate	CSF	Increase	A slight increase compared to non-injured controls in moTBI and sTBI patients	Glenn et al. [37]
	Lactate	CSF	Increase	Significantly increased compared to control and survival groups	Stefani et al. [38]
	Lactate	MR Images	Increase	Diffusely high signal of lactate resonance intensity in patients with acute TBI	Marino et al. [25]
Organooxygen compounds					
	Inositol	Serum/Plasma/Microdialysate	Increase	Found to increase in patients with detectable pathology on a CT scan or the presence of a mass lesion	Dickens et al. [36]
	(2,3-BPG)	Serum/Microdialia	Increase	Found upregulated in sTBI patients and a strong association with TBI severity, roughly a 100-fold upregulation compared to controls	Oresic et al. [33]
	Propylene glycol	CSF	Increase	Significantly higher concentrations in moTBI and sTBI patients	Glenn et al. [37]
	Gluconate	Serum/Plasma	Decrease	TBI blood had less than or equal to 60% the concentration of HC in arterial plasma	Wolahan et al. [34]
	Glucose-6-phosphate	Serum/Plasma	Decrease	Significant decrease in jugular venous blood from arterial levels	Wolahan et al. [34]
	Glycerol	Microdialysis	Increase	Significant positive conc. correlations between extracellular NAA and glycerol of 8 patients	Shannon et al. [28]
	Ribonic acid	Serum/Plasma	Increase	Found to increase in patients with detectable pathology on a CT scan or the presence of a mass lesion following TBI	Dickens et al. [36]
	Kynurenine	CSF	Increase	Displayed median levels similar to control days 0–3, but at days 4 and 5 showed significant elevation over controls post sTBI	Yan et al. [62]
	Myo-inositol	Serum/Plasma	Increase	Found elevated in TBI patients and proportional to differing severity	Thomas et al. [32]
Organic carbonic acids and derivatives					
	Urea	Serum/Plasma	Decrease	Significant decrease in plasma of sTBI patients compared to healthy volunteers, orthopedic controls, and mTBI patients	Jeter et al. [26]
Quinolines and derivatives					
	Kynurenic acid	CSF	Increase	Increased post sTBI compared to controls and reached a plateau after day 2 which lasted until day 5	Yan et al. [62]
Pyridines and derivatives					
	Niacinamide	Serum/Plasma	Decrease	TBI blood had less than or equal to 60% the concentration of HC in jugular blood	Wolahan et al. [34]
	Quinolinic acid	CSF	Increase	Concentration significantly increased between days 1 and 5 compared to controls	Yan et al. [62]
Imidazopyrimidines					
	Xanthine	Serum/Plasma	Increase	Significant net cerebral release or increase in jugular venous blood	Wolahan et al. [34]
	Xanthine	Urine	Increase	Increased levels following recovery	Bykowski et al. [30]
	Hypoxanthine	Urine	Increase	Found increased following recovery	Bykowski et al. [30]
Diazines					
	Thymine	Urine	Increase	Significant negative correlation (increase) to patients decreasing GCS scores	Bykowski et al. [30]
Purine nucleosides					
	Adenosine	Urine	Increase	Found to be significantly upregulated over recovery	Bykowski et al. [30]
	Inosine	Urine	Increase	Found upregulated in urine	Bykowski et al. [30]
	Deoxyinosine	Urine	Increase	Found upregulated after recovery	Bykowski et al. [30]
	Guanosine	Urine	Increase	Found upregulated in urine	Bykowski et al. [30]
Indoles and derivatives					
	Indole-3-propionic acid	Serum/Microdialysis	Decrease	Found downregulated in all TBIs, more strongly in sTBI	Oresic et al. [33]
	Tryptophan	CSF	Increase	Increased in CSF compared to controls between days 0 to 5 in sTBI, however, median concentrations did not change much	Yan et al. [62]
	Tryptophan	Plasma/Serum	Decrease	Decreased post sTBI in serum from days 0 to 4	Yan et al. [62]
Organonitrogen compounds					
	Choline	Serum/Plasma	Decrease	Significantly reduced compared to mTBI and healthy volunteers	Dash et al. [29]
	Choline	Serum/Plasma	Increase	Net cerebral release or increase in jugular venous blood	Wolahan et al. [34]
	Choline	MR Imaging	Increase	From patients with differing degrees of TBI including sTBI	Marino et al. [25]
	Spermidine	Serum/Plasma	Decrease	Significantly lower levels post moTBI and sTBI	Huang et al. [65]

*CSF* cerebrospinal fluid; *CT* computed tomography; *GCS* Glasgow Coma Scale; *HC* healthy controls; *HMDB* Human Metabolome Database; *mTBI* minor traumatic brain injury; *moTBI* moderate traumatic brain injury; *NAA* N-acetylaspartate; *sTBI* severe traumatic brain injury; *TBI* traumatic brain injury

### Pathway analysis revealed affected glycine and serine metabolism, branched chain amino acid metabolism, glycolysis, and several other amino acids metabolisms

**Fig. 2:**
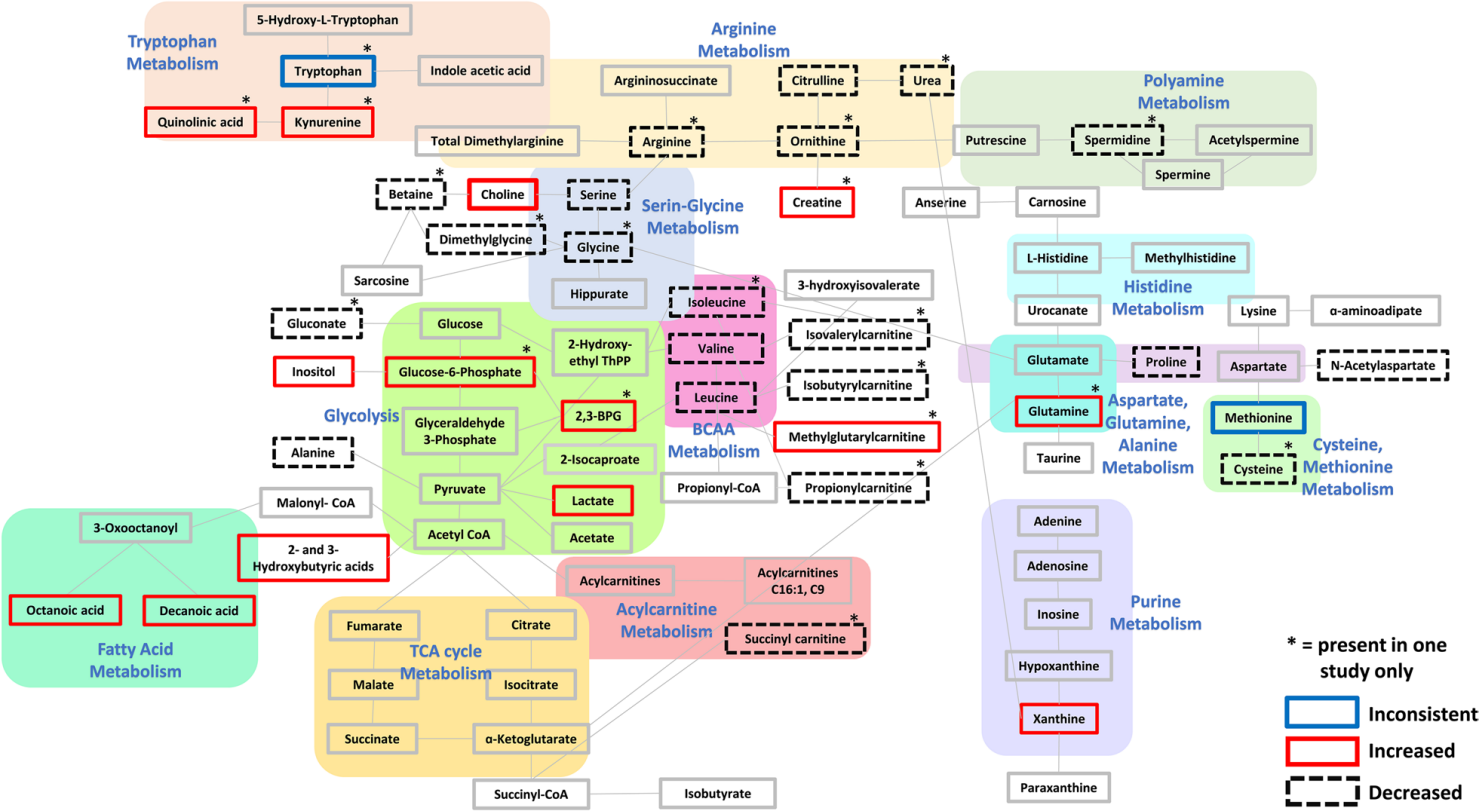
Pathway analysis diagram generated manually using metabolites identified in the reviewed literature and the KEGG Pathway Database. Pale colored boxes represent metabolic pathways. Pathways are simplified for summary purposes. Increased metabolites found in reviewed studies are indicated by a thick red outline and decreased metabolites are indicated by a thick black dashed outline. Metabolites with conflicting results are indicated by a dark blue outline. An *asterisk* (*) was placed on each metabolite that was found in only one of the reviewed studies. All other highlighted metabolites without an *asterisk* were present in more than one reviewed study and encompass the fifteen ‘recurrent’ metabolites identified in this review

Further exploration of the collected metabolic data led to the manual generation of a metabolic pathway analysis diagram using the KEGG Pathway Database (Fig. 2). The pathway analysis diagram demonstrated significantly affected glycine and serine metabolism, with most metabolites displaying a decrease in concentration following sTBI. Furthermore, BCAA metabolism was significantly affected, attributable to a decrease in most metabolites involved. Glycolysis was also significantly affected, displaying an increase in most metabolites. Fatty acid metabolism was also affected due to an increase in octanoic and decanoic acids. The amino acids with the most altered metabolic pathways appeared to be tryptophan, which exhibited an increase in most of the related metabolites, and arginine, which exhibited a decrease in most of the related metabolites. Cysteine and methionine metabolism were also affected, displaying a general decrease in most metabolites. Only a few metabolites were identified for polyamine metabolism, acylcarnitine metabolism, and purine metabolism. TCA cycle metabolism displayed no affected metabolites following sTBI in the collected data.

## Discussion

The results of this scoping review suggest several metabolites with biomarker potential for sTBI in adults discovered through a review of the current primary literature. However, it is important to consider the role that sample origin may play in the significance of metabolite data. This review included publications with several sample origins since different biological sources can be applied for metabolic analysis. Sample origin should be considered when interpreting the data, as biological sources more closely related to the site of injury (the brain) give more precise measurements of local metabolic changes. It is also important to consider which sample type is most accessible for each severity of TBI. Serum and plasma are typically the easiest to collect from all TBI patients, while the methods of retrieving CSF and microdialysate are better suited for sTBI patients, who may already be sedated and instrumented. Due to sampling location, CSF and microdialysate also provide a more accurate depiction of how metabolites surrounding the brain are affected. Despite this, serum and plasma were the most common biological sources used by the publications in this review, followed by CSF and microdialysate. Urine and MRIS were also utilized in some of the publications, however, conflicting results have been found for urine in the past, and MRIS can only provide a very limited resolution or area of brain assessment to date [23].

To understand TBI pathophysiology, it is important to consider the mechanisms by which the brain injury occurs and how metabolite alteration could affect the progression and severity of sTBI. TBI is widely understood as a consequence of both primary injuries; damage occurring at the moment of injury, such as an impact or penetration, and secondary injuries; widespread damage produced hours or days after injury due to a cascade of cellular and inflammatory processes [39, 40]. Familiar secondary injury mechanisms include hypotension [11], hypoxemia [10, 11], ischemia potentially caused by a hypermetabolic surge [8], excitotoxicity [29], and oxidative stress [29, 41]. These secondary injuries could be caused by the metabolic and biochemical changes that occur in the brain following a primary insult, such as those identified in this review.

In the brain, amino acids play an important role in the synthesis of small-molecule neurotransmitters. Figure 2 displays the significant changes in glycine and serine metabolism attributable to sTBI. Glycine is the main inhibitory neurotransmitter in the brainstem and spinal cord and is metabolized from L-serine, which binds to glycine receptors. Serine also plays a significant role in the brain, as it acts as a neuromodulator and neurotransmitter under different conditions. This study identified a recurrent decrease in serine following sTBI, but only one study reported a decrease in glycine [25]. Serine plays a neuroprotective role in the CNS by decreasing neurotoxicity through the activation of glycine receptors and reducing inflammation by lowering proinflammatory factors [42]. Therefore, a decrease in serine could lead to enhanced inflammatory responses and cerebral ischemia following a head injury [43]. A recent study injecting L-serine into rats sustaining a TBI found decreased neurological deficit scores, decreased neuron loss, and overall greater neuroprotection following L-serine injection [44].

The metabolism of arginine, another essential amino acid, was also affected following sTBI (Fig. 2). One biological function of arginine is to serve as a precursor of nitric oxide, which has many physiological functions in the brain, such as protection against further brain injury [45, 46]. Therefore, a reduction in arginine and related metabolites is likely unfavorable to sTBI patients. Arginine participates in the synthesis of creatine, and a deficiency of creatine is associated with many neurological conditions including speech impairments [45, 47]. Valine, leucine, and isoleucine are all BCAAs in a close relationship with aromatic amino acid catabolism, which produces brain neurotransmitters such as serotonin, dopamine, and norepinephrine [48]. In this review, two common BCAAs, valine and leucine, were found to decrease following sTBI. Valine and leucine are both important amino acids in the compartmentalization of glutamate [48], which is a primary excitatory neurotransmitter commonly found in excess after TBI and can cause a secondary injury known as glutamate excitotoxicity [49]. Glutamate excitotoxicity leads to cell apoptosis and neuronal death, which could be responsible for reduced cognitive function [50]. Figure 2 shows significantly affected BCAA metabolism, with nearly all metabolites showing a reduction following sTBI. Alanine is synthesized by BCAAs (valine, leucine, and isoleucine) and was also found in this review to be decreased. Experimentally, BCAAs are known to carry a neuroprotective role and contain neurorestorative properties when supplemented post injury [27, 51, 52]. Therefore, a reduction in BCAAs may contribute to diminished neuroprotection and a high susceptibility to glutamate excitotoxicity.

Glycolysis is a fundamental process by which the body produces energy, both aerobically and anaerobically. Disruptions in cerebral oxidative metabolism can have significant impacts on the recovering brain and have been correlated with poor long-term outcomes such as vegetative state and death [53, 54]. Several of the primary metabolites involved in glycolysis metabolism (see Fig. 2), such as lactate and inositol, were found to be increased following sTBI in this review. An increase in anaerobic glycolysis suggests a higher rate of glucose being metabolized per mole of oxygen through anaerobic mechanisms, which could be considered as the injured brain entering a state of ‘hyperglycolysis’ [54, 55]. A surge in lactate among sTBI patients, as seen in this review, supports the notion of increased anaerobic glycolysis energy metabolism [56]. Lactate has been interpreted in TBI as both (1) a therapeutic option to compensate for decreased cerebral metabolic rate [57] and cognitive impairment [58] and (2) as potentially harmful in having associations with hypoxia and mitochondrial dysfunction [59].

A secondary component of this study included the classification of metabolites designated by the Human Metabolome Database. Exceptions found within some of the categories indicate the large lack of uniformity across current metabolomic studies. The most significant findings from this section of the analysis were an increase in hydroxy acids and a general decrease in carboxylic acids following sTBI. Carboxylic acids and their derivatives encompass numerous amino acids, including serine, alanine, tyrosine, asparagine, threonine, the BCAAs and other closely related derivatives such as gamma-glutamylvaline and citrulline. The overarching similarity between many of the carboxylic acids is the role they may play in excitotoxicity and biochemical alterations which cause a change in regular homeostatic levels of the brain. The altered metabolites in the hydroxy acids and derivatives class included increased 2/3-hydroxybutyric acid and lactate. Increased 2/3-hydroxybutyric acid is associated with poor outcomes and could be connected to ketogenic metabolism, where the increased presence of ketone bodies may be responsible for increasing blood–brain barrier permeability [60]. Deeper understanding of the affected classes involved in sTBI pathology could aid in the potential creation of specialized therapeutics, and thus require further research.

Methionine is an important precursor for glutathione, an antioxidant molecule that works to reduce the stress caused by oxidative damage, therefore, a decrease in methionine could allow for elevated oxidative damage [29]. Contradicting results were found for methionine levels post sTBI in this review. Increases in urine methionine levels were only found in one study [30], however, urine is not as reliable as other biological sources of metabolites, and supporting literature primarily points towards a decrease in methionine leading to poorer clinical outcomes. The tricarboxylic acid (TCA) cycle is an ascertained affected pathway in the progression of secondary injury in sTBI, however, surprisingly, no significant recurrent changes were identified in the TCA cycle metabolites from the reviewed literature (Fig. 2). This could be due in part to the time of sample collection after the head injury, as it is unclear if cerebral TCA metabolism changes over time following sTBI. Interestingly, other omics-type studies show a reduction in TCA protein gene expression, and alterations in TCA enzymes have been seen in closed-head impact mouse models post sTBI [61].

### Limitations

The complex nature of TBI, distinguishing between primary and secondary brain injury, the confounding effects of polytrauma, and the potential effects of medications and feeding (nutrition) on metabolomics in patients with sTBI interfere with determining a precise metabolomic profile for sTBI using metabolomics, especially when examining metabolite changes from the reviewed literature. While this study produced several sufficiently supported metabolites changes prevalent in sTBI research, it is too early to determine whether these metabolites can be used clinically as biomarkers for sTBI until controlled validation studies are completed. Comparing clinical findings across a multitude of studies evokes numerous potential confounders and difficulties. As mentioned, the differences in sampling methods—CSF in comparison to serum/plasma and urine, and even venous blood compared to arterial blood—can account for large ambiguities in metabolite concentrations across the datasets. Another large confounder is the time a sample was collected (2 h. post sTBI vs. 6d post sTBI) which affects metabolite concentration and cannot be easily accounted for in this study. Further, there is no reason to assume that metabolomic changes in mTBI are like those seen in sTBI. While this study aimed to specify sTBI data collected from adults, several studies included patients just over the age of 16 or combined varying degrees of TBI in their data. Furthermore, data that was not verifiable or entirely discernible was not included, potentially leading to the dismissal of important metabolite changes and publications. Thus, many confounders, including those mentioned above, undoubtedly had an affect on the results presented here.

## Conclusion

This scoping review sought to identify commonalities between the published primary literature investigating metabolomics in sTBI to determine if a reliable set of metabolites (‘biomarkers’) or a metabolic profile could be determined that may be of clinical use. This study adds to the current knowledge on metabolomics in sTBI by summarizing and compiling the recent literature to determine potentially clinically relevant biomarkers. To our knowledge, a scoping review as comprehensive as that presented here, has not been completed to date, likely due to the large variability between metabolomics studies in sTBI. While identifying key metabolites between studies proved challenging and potentially problematic, fifteen ‘recurrent’ metabolites, several HMDB classes, and their affected pathways were identified in this review. Furthermore, these metabolites and their pathways were supported by suggesting potential secondary mechanisms of injury, such as oxidative damage and excitotoxicity caused by the alterations in metabolite concentrations following sTBI. This study recognizes several metabolites with biomarker potential; however, it is clear that further studies are needed to determine the significance and useability of these findings. Once that is achieved, more specialized therapeutics could be designed to slow or alter the mechanisms by which sTBI causes injury, potentially decreasing the detrimental effects of sTBI overall.

### Supplementary Information

**Additional file 1: Table S1.** Summary table of eligible publications exploring metabolomics in severe traumatic brain injury following selection process.

## Data Availability

All data generated or analyzed during this study are included in this published article [and its supplementary information files].

## Abbreviations

^1^H-MRIS: Proton nuclear magnetic resonance imaging
^1^H-NMR: Proton nuclear magnetic resonance
^31^P-MRS: Phosphorous magnetic resonance spectroscopy
BCAA: Branched chain amino acid
BDNF: Brain-derived neurotrophic factor
CSF: Cerebrospinal fluid
CT: Computed tomography
GC–MS: Gas chromatography-mass spectrometry
GC-QTOFMS: Gas chromatography coupled to quadrupole time-of-flight mass spectrometry
GCxGC-MS: 2D gas chromatography coupled to mass spectrometry
GCxGC-TOFMS: 2D Gas Chromatography coupled to Time-Of-Flight Mass Spectrometry
GDNF: Glial cell-derived neurotrophic factor
HMBD: Human metabolome database
HPLC: High-performance liquid chromatography
KEGG: Kyoto encyclopedia of genes and genomes
LC–MS: Liquid chromatography-mass spectrometry
LC-QTOFMS: Liquid Chromatography Coupled to Quadrupole Time-Of-Flight Mass Spectrometry
moTBI: Moderate traumatic brain injury
MRI: Magnetic resonance imaging
MRIS: Magnetic resonance imaging spectroscopy
mTBI: Minor traumatic brain injury
NAA: N-acetylaspartate
sTBI: Severe traumatic brain injury
TBI: traumatic brain injury
UV: Ultraviolet
