# Supplementary material for: Metabolomics in severe traumatic brain injury: a scoping review
Source: BMC Neurosci. 2023 Oct 16;24:54. doi: 10.1186/s12868-023-00824-1 (PMC10577974; doi:10.1186/s12868-023-00824-1)
Supplement: Supplementary file 1 — Additional file 1: Table S1. Summary table of eligible publications exploring metabolomics in severe traumatic brain injury following selection process. [file 12868_2023_824_MOESM1_ESM.pdf]

Table A1. Summary table of eligible publications exploring metabolomics in severe traumatic brain injury following selection process

Year of Study	Sample Type	Sample Size	Detection Technique	Study setting	Refs.
(2006)	MRI	TBI patients=10, normal controls=10	^{1}H-MRSI	^{1}H-MRSI examinations on patients to determine metabolite levels of N-acetyl aspartate, choline, creatine, and lactate post TBI	Marino et al. (25)
(2011)	Serum/Plasma	sTBI patients=19, healthy contols=44	HPLC	Measured the effects of changes in branched-chain amino acids and plasma aromatic amino acid levels	Vuille-Dit-Bille et al. (35)
(2012)	Serum/Plasma	sTBI patients=20, mTBI patients=18, Orthopedic injury patients=15, Healthy volunteer=20	LC-MS/GC-MS	Measured plasma levels of L-arginine and similar key metabolic products in patients post TBI and compared them to healthy volunteers and orthopedic controls	Jeter et al. (26)
(2013)	Serum/Plasma	sTBI patients=20, mTBI patients=18, Orthopedic Injury=15, Healthy volunteer=20	LC-MS/GC-MS	Identified altered metabolic cascades and branched-chain amino acids in acute sTBI and mTBI patients	Jeter et al. (27)
(2013)	CSF	TBI patients (moTBI or sTBI only, 14 yrs or older)=44, non-injured controls=13	^{1}H-NMR	Analyzed metabolites from the CSF of patients following TBI	Glenn et al. (37)
(2015)	CSF	sTBI patients=28, CSF control patients=11, serum control patients=20	HPLC/GC-MS	Investigated the effect of the kynurenine pathway on brain damage post TBI	Yan et al. (62)
(2016)	Serum/Plasma	sTBI patients=20, mTBI patients=20, healthy volunteers=20 (ages ranged from 14 to 57)	LC-MS/GC-MS	Specifically examined methionine alteration and related metabolites following TBI	Dash et al. (29)
(2016)	Serum/ Microdialysate	TBI patients=144 (mTBI=108, moTBI=14, sTBI=22), acute orthopedic non-brain injury controls=28, validation study=67 TBI (mTBI=37, moTBI=7, sTBI=22, acute orthopedic non-brain injury controls=27	GCxGC-TOFMS	Aimed to determine markers and outcome prediction for TBI patients by evaluating two independent cohorts using metabolic profiling. Combined results with clinical and CT data	Oresic et al. (33)

(2016)	Microdialysate	sTBI patients=30, non-TBI neurosurgical patients =6	HPLC	Analyzed the behaviour of extracellular NAA levels post sTBI	Shannon et al. (28)
(2017)	CSF	sTBI patients=20 (divided into non-survival=6 & survival=14), healthy controls=20	HPLC	Assessed levels of glutamate, lactate, BDNF, and GDNF in sTBI patients between 2 and 4 h post injury to determine biomarker potential	Stefani et al. (38)
(2018)	Serum/Plasma/ Microdialysate	TBI patients=144 (sTBI patients=22, moTBI patients=14, mTBI patients=108), orthopedic injury controls=55, validation cohort=66 (sTBI=23, moTBI=7, mTBI=36)	GCxGC-TOFMS/GCxGC-MS	Explored if metabolites could validate the need for CT scans in TBI patients as well as predict the nature of brain injury	Dickens et al. (36)
(2019)	Serum/Plasma	sTBI patients=26, healthy controls=6	LC-MS	Compared metabolite levels in arterial and jugular venous samples to determine changes post sTBI and study the effects on cerebral metabolism	Wolahan et al. (34)
(2019)	Microdialysate	TBI patients=26, validation cohort=12	LC-MS	Combined clinical and cerebral microdialysis metabolomics data to investigate metabolites and associations with patient outcomes	Eiden et al. (63)
(2020)	Serum/Plasma	TBI patients=96 (mTBI=79, moTBI=10 sTBI=7)	GCxGC-TOFMS	Related circulating metabolites to findings from structural MRI post TBI	Thomas et al. (64)
(2020)	Serum/Plasma	TBI patients=29 (sTBI=12, moTBI=8, mTBI=9), control patients=10	HPLC with UV-visible detection	Investigated the therapeutic potential of spermidine in TBI in humans and mouse models	Huang et al. (65)
(2021)	MRI	sTBI patients=10	^{31}P-MRS	Tested the diagnostic potential of ^{31}P-MRS to detect cerebral energy metabolism changes post TBI	Pinggera et al. (66)
(2021)	MRI	sTBI patients=26, control patients=20	^{31}P-MRS	Performed 31P-MRS post TBI at two separate time intervals to detect alterations in cerebral energy metabolism	Pinggera et al. (67)
(2021)	Urine	TBI patients=8 (sTBI=2, moTBI=3, mTBI=3), no controls,	^{1}H-NMR	Analyzed urine samples in TBI patients to determine if metabolites changed during	Bykowski et al. (30)

		but incorporated a within-subject control design		recovery, correlated to injury severity, and reflected TBI mechanisms	
(2022)	Serum/Plasma	TBI patients (sTBI and moTBI only)=51	LC-MS	Investigation of serum glycome patterns following TBI to determine glyco-biomarker signatures and clinical relevance	Mondello et al. (31)
(2022)	Serum/Plasma	TBI patients=716, non-TBI patients=229 (orthopedic controls=40, internal medicine=96, other neurological patients=93). Samples collected from the Center-TBI, validation dataset=558 TBI patients	GC-QTOFMS/LC-QTOFMS	Intended to define and describe the metabolome related to acute TBI and investigate its effect on severity and patient outcome post TBI using metabolomics and lipidomics	Thomas et al. (32)

Note. Abbreviations: ^{1}H-MRSI, proton nuclear magnetic resonance imaging; ^{31}P-MRS, phosphorous magnetic resonance spectroscopy; BDNF, brain-derived neurotrophic factor; CSF, cerebrospinal fluid; CT, computed tomography; GC-MS, gas chromatography-mass spectroscopy; GC-QTOFMS, gas chromatography coupled to quadrupole time-of-flight mass spectrometry; GCxGC-MS, 2D gas chromatography coupled to mass spectrometry; GCxGC-TOFMS, 2D gas chromatography coupled to time-of-flight mass spectrometry; GDNF, glial cell-derived neurotrophic factor; ^{1}H-NMR, proton nuclear magnetic resonance; HPLC, high performance liquid chromatography; LC-MS, liquid chromatography-mass spectrometry; LC-QTOFMS, liquid chromatography coupled to quadrupole time-of-flight mass spectrometry; moTBI, moderate traumatic brain injury; MRI, magnetic resonance imaging; mTBI, minor traumatic brain injury; NAA, acetylaspartate; sTBI, severe traumatic brain injury; TBI, traumatic brain injury; UV, ultraviolet.
